# Tribological properties of attapulgite/La_2_O_3_ nanocomposite as lubricant additive for a steel/steel contact

**DOI:** 10.1039/c8ra02835d

**Published:** 2018-05-09

**Authors:** Feng Nan, Kaihe Zhou, Shuan Liu, Jibin Pu, Yunhui Fang, Wenxuan Ding

**Affiliations:** Key Laboratory of Marine Materials and Related Technologies, Zhejiang Key Laboratory of Marine Materials and Protective Technologies, Ningbo Institute of Materials Technology and Engineering, Chinese Academy of Sciences Ningbo 315201 China nanfeng2005@126.com pujibin@nimte.ac.cn +86 17855849895; State Grid Zhejiang Electric Power Supply Company Ningbo Power Supply Company Co., Ltd Ningbo 315201 China

## Abstract

Attapulgite is a layered silicate with good friction-reduction and self-repairing properties. In order to further improve its tribological properties, the present work mainly focuses on the preparation of attapulgite/La_2_O_3_ nanocomposite and study on its tribological behaviors. The tribological properties of mineral lubricating oil (150SN) containing attapulgite/La_2_O_3_ nanocomposite were investigated through an Optimal SRV-IV oscillating friction and wear tester. The rubbing surfaces and generated tribofilms were characterized by SEM, EDS, XPS and nanoindentation. Results indicated that the friction-reducing ability and antiwear property of the oil were both remarkably improved by attapulgite/La_2_O_3_ nanocomposite. A tribofilm mainly composed of Fe, Fe_3_C, FeO, Fe_2_O_3_, FeOOH, SiO, SiO_2_ and organic compound was formed on the rubbing surface under the lubrication of oil with attapulgite/La_2_O_3_ nanocomposite. The tribofilm possess excellent self-lubricating ability and mechanical properties, which is responsible for the reduction of friction and wear.

## Abstract 2

Attapulgite is a layered silicate with good friction-reduction and self-repairing properties.

## Graphical abstract

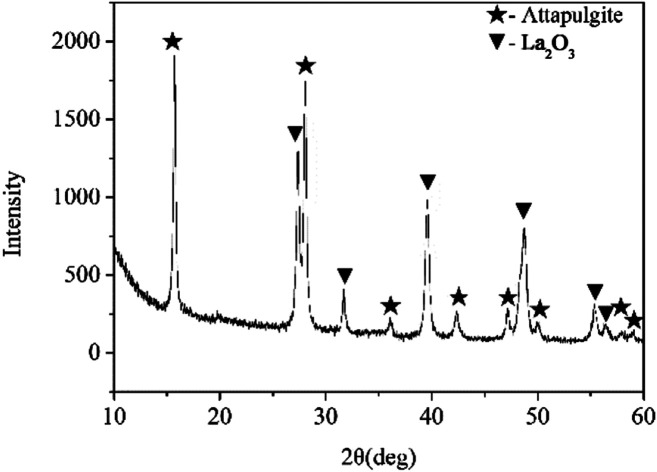

## 1. Introduction

Wear caused by friction is one of the main causes of materials failure. It is generally known that employing ultrafine powders as lubricant additives is effective in reducing friction and wear.^1–5^ In recent years, some researchers have reported that several layer-chained silicates, such as serpentine and attapulgite, can be used as lubricant additives.^6–10^

Zhang *et al.*^6,7^ investigated the friction and wear properties of surface-coated natural serpentine powders as lubricant additives on a steel–steel contact by Optimal SRV oscillating friction and wear tester. The friction coefficient and wear loss of the base oil (CD 15w-40) were both obviously decreased in the effect of serpentine powders. During the friction procedure, tribochemical reactions occurred between serpentine particles and friction surfaces, consequently an amorphous tribofilm enriched in Si–O structures was formed on the rubbing surface. The tribofilm was mainly composed of iron oxides, silicon oxides, graphite and organic compounds. The tribofilm possess excellent lubricating ability and mechanical properties, which is responsible for the reduced friction and wear. Yu *et al.*^8^ investigated the tribological behavior of natural serpentine mineral powders as lubricant additive through CETR UMT-2 test system. It is found that the lubricity of the base oil (500SN) was improved remarkably by serpentine mineral powders. A nanocrystalline tribofilm, mainly composed of Fe_3_O_4_, FeSi, SiO_2_, AlFe and Fe–C compound (Fe_3_C), was formed on the worn surface under the lubrication of 500SN oil with serpentine powders. The tribofilm possess a high surface hardness (about 8.0 GPa) and a low modulus (<240 GPa). Qi *et al.*^9^ investigated the friction and wear behaviors of nanoscale serpentine and heat-treated serpentine as lubricating oil additives at 400 °C. In the effect of two kinds of lubricant additives, self-repairing protective layers could be well formed on the contact surfaces. During the friction procedure, tribochemical reactions and metallurgical bonding are the dominant mechanisms.

As we can see, the research on serpentine powders as lubricant additives is systematic and insightful. However, there were very few reports of attapulgite powders using as lubricant additives. Attapulgite is a kind of layer-chained silicates that possess similar chemical composition and crystal structure with serpentine. In the previous study, we found that attapulgite powders can also improve the friction-reducing ability and antiwear property of lubricating oil. With the lubrication of oil with attapulgite powders, a complex tribofilm, mainly composed of FeO, Fe_2_O_3_, FeOOH, SiO_*x*_ and organic compound formed on the worn surface. But the improvement effect of attapulgite powders is not ideal, especially at low load and high load. This result demonstrated that the tribofilm forming ability of attapulgite powders is poor and the properties of formed tribofilm are poor. In addition, the friction-reducing and antiwear mechanisms of the attapulgite are still obscure.

Rare-earth oxides possess some special properties, including adsorbability and catalytic activity.^11,12^ And some researches had reported the using of La_2_O_3_ in the researches field of tribology. Xu *et al.*^13^ have investigated the tribological properties of La_2_O_3_ nanoparticles as lubricant additives in bio formulated diesel. It was found that with addition of 1.0 wt% La_2_O_3_ nanoparticles, friction and corrosive wear were obviously reduced. The effect of nano-bearing was proposed as the reason for the reduction of friction and wear. Mo *et al.*^14^ investigated the sliding friction and wear behaviors of Cu–La_2_O_3_-graphite composites against Cu–Ag alloy. The results showed that the hardness, flexural strength, wear resistance and electrical resistivity of Cu-graphite composites were increased with the addition of La_2_O_3_.

In order to improve the friction-reducing and antiwear properties of attapulgite powder, La_2_O_3_ were selected as repairing accelerant. In this work, the tribological behaviors of attapulgite/La_2_O_3_ nanocomposite as lubricant additives were investigated using an optimal SRV-IV oscillating friction and wear tester. The microstructure, chemical composition and mechanical properties of the tribofilm generated during the friction procedure were characterized. The strengthening mechanism of La_2_O_3_ was discussed.

## 2. Experimental details

### 2.1 Material processing and sample preparation

Attapulgite powder was purchased from Jiangsu Jiuchuan Nanometer material Science and Technology Ltd, China. La_2_O_3_ powder was purchased from Beijing DK Nano Technology Co., Ltd, China. 150SN was purchased from Qingdao Compton Technology Co., Ltd, China. Oleic acid was supplied by Aladdin Co., Ltd, China. All materials were used without any treatment.

The preparation process of oil containing attapulgite/La_2_O_3_ nanocomposites is described as follows. First, 6.0 g attapulgite powder with 0.0 g, 2.0 g, 4.0 g, 6.0 g and 8.0 g La_2_O_3_ powder were carefully dissolved in 100 ml ethanol containing 5.0 ml oleic acid respectively. Second, the mixed solutions were milled by in a ball crusher. The rotational speed of ball crusher is 250 rpm and the milling duration is 8 h. Third, the mixed solutions were heated in a vacuum drying oven to remove ethanol, and then the attapulgite/La_2_O_3_ nanocomposites were prepared. Finally, specific amount of attapulgite/La_2_O_3_ nanocomposites were added into the 150SN base oil and subsequently were treated by ultrasound for 60 minutes. Lubricants of different components shown in Table 1 were prepared.

**Table 1:** The lubricants prepared in this work

Code name	Constituent
L1	150SN
L2	150SN + 0.6 wt% attapulgite
L3	150SN + 0.6 wt% attapulgite + 0.2 wt% La_2_O_3_
L4	150SN + 0.6 wt% attapulgite + 0.4 wt% La_2_O_3_
L5	150SN + 0.6 wt% attapulgite + 0.6 wt% La_2_O_3_
L6	150SN + 0.6 wt% attapulgite + 0.8 wt% La_2_O_3_
L7	150SN + 0.4 wt% La_2_O_3_

### 2.2 Friction and wear tests

The tribological properties of the lubricants were investigated using an optimal SRV-IV tribo-tester with a ball-on-disk configuration as shown in ref. 15. The balls with a diameter of 10.0 mm and hardness of HV 710 were made of AISI 52100 steel. The disks with a diameter of 24.0 mm, a thickness of 8 mm and hardness of HV 710 were made of AISI 1045 steel. And the surfaces of the disks were mechanically ground and polished to a minute surface (*R*_a_ ≈ 0.2 μm).

In the friction and wear tests, the optimum addition of attapulgite/La_2_O_3_ nanocomposites was investigated firstly. Subsequently, the effect of load and frequency on the tribological behaviors of the nanocomposites was investigated. Detailed test parameters were shown in Table 2. For each experimental condition, each test was carried out for three times. The average friction coefficient was calculated during the steady friction state. A MicroXAM 3D non-contact surface mapping profiler was employed to characterize the rubbing surfaces and measure the volumes of the wear scar on the disks. Each wear scar was measured for three times and the average value was calculated. At last, the wear rate was calculated. The wear rate was defined as the wear volume per unit product of sliding distance and load.

**Table 2:** The experimental parameters of the tribological tests

	Load (*N*)	Contact pressure (GPa)	Sliding frequency (Hz)	Temperature (°C)	Amplitude (mm)	Test duration (min)	Sliding distance (m)
Component optimization	60	1.83	30	30	1	60	216
Load effect	20	1.29	30				216
	40	1.62					
	60	1.83					
	80	2.04					
	100	2.17					
Frequency effect	60	1.83	10				72
			20				144
			30				216
			40				288
			50				360

### 2.3 Characterization

The XRD pattern of attapulgite/La_2_O_3_ nanocomposite was analyzed by an Advanced D8 diffractometer. The morphology of attapulgite/La_2_O_3_ nanocomposite was examined by scanning electron microscopy (Hitachi S4800) and transmission electron microscopy (Tecnai F20).

The morphologies and element distribution of the rubbing surfaces were characterized by SEM (NovaNano SEM 650) equipped with EDS (Oxford). The chemical states of some typical elements were characterized by XPS (ESCALAB 250Xi). Monochromatic AlK_α_ X-ray radiation (1486.6 eV) was used as the excitation source of XPS. The XPS spectra were calibrated with reference to the C1s line at 284.8 eV. The nano-hardness and elastic modulus of the metal matrix and tribofilms were detected by the nano-indentation tester (G200 Nano Indenter). The indentation controls displacement from 100 nm to 500 nm at a single point. And the variations of nano-hardness and elastic modulus with depth were recorded.

## 3. Results and discussion

### 3.1 Characterization of the nanocomposite

**Fig. 1:**
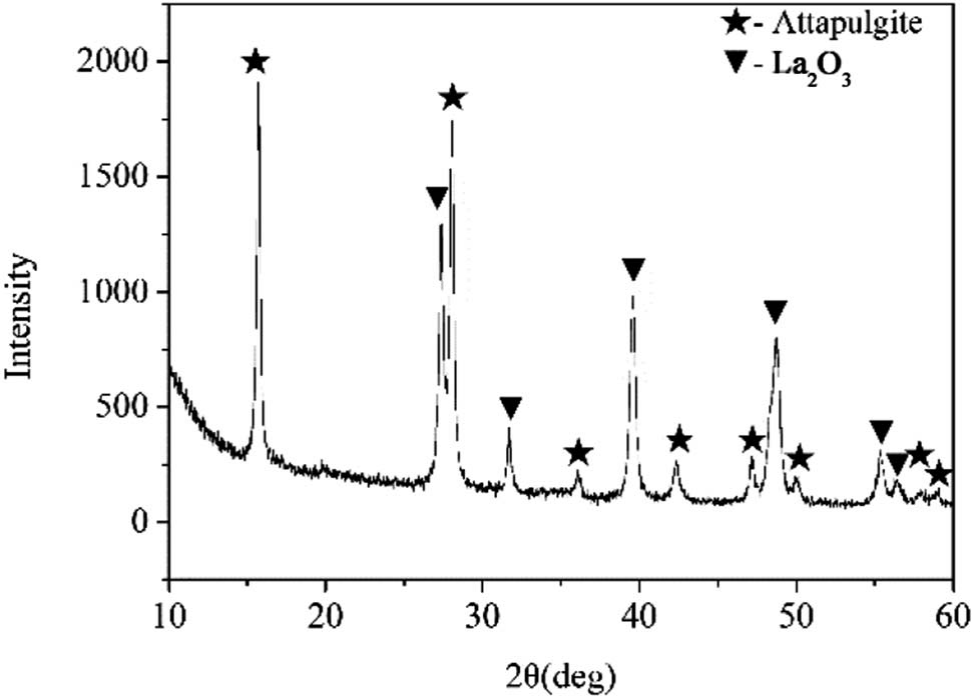
XRD pattern of the attapulgite/La_2_O_3_ nanocomposite containing 0.4 wt% La_2_O_3_.

**Fig. 2:**
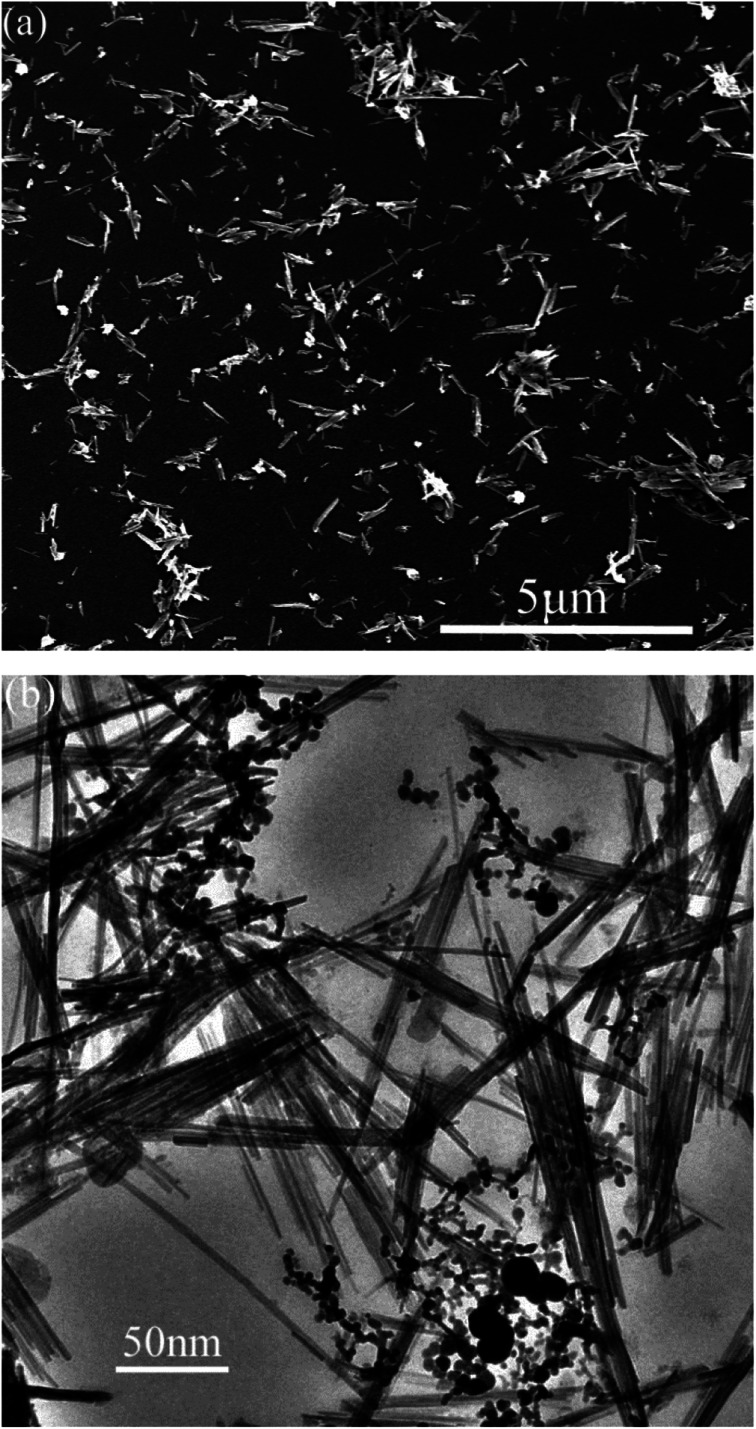
(a) SEM and (b) TEM morphology images of the attapulgite/La_2_O_3_ nanocomposite containing 0.4 wt% La_2_O_3_.

Fig. 1 shows the XRD pattern of attapulgite/La_2_O_3_ nanocomposite. The mass ratio of attapulgite and La_2_O_3_ is 3 : 2. On the XRD pattern, only typical peaks of attapulgite and La_2_O_3_ were detected. All peaks were sharp and intense. The result indicated that attapulgite and La_2_O_3_ was successfully incorporated together. Fig. 2 shows the SEM and TEM morphology images of attapulgite/La_2_O_3_ nanocomposite. The SEM image shown in Fig. 2(a) indicated that the as-prepared nanocomposite present good dispersion in organic medium; no aggregates with large size had been founded. The TEM image shown in Fig. 2(b) indicated that most spherical La_2_O_3_ nanoparticles were absorbed on the surface of attapulgite nanofibers.

During the ball milling process, the agglomerated nanoparticles were scattered by mechanical force. And the grafting of oleic acid molecule remarkably improved the lipophilicity of nanoparticles, effectively preventing the reunion of the nanoparticles. Consequently, the nanoparticles could gain long term stability in the oil. In the meantime, attapulgite nanofibers and La_2_O_3_ nanoparticles can combine together through the adsorption of attapulgite and hydrogen bonding. For the as-prepared lubricants, after being settled for 30 days, no obvious settlement was observed. In addition, with the addition of attapulgite/La_2_O_3_ nanocomposite, the typical physicochemical properties of the 150SN were not changed basically, which can be seen from Table 3.

**Table 3:** Typical physicochemical properties of the oil and oil containing attapulgite/La_2_O_3_ nanocomposite

	Item value (ASTM)				
	Density (g cm^−3^)	Kinematic viscosity (mm^2^ s^−1^)	Viscosity index	Pour point (°C)	Flash point (°C)
150SN	0.877	5.32, 100 °C/31.7, 40 °C	118	−15.5	220
150SN containing attapulgite/La_2_O_3_ nanocomposite	0.883	5.28, 100 °C/31.5, 40 °C	119	−14.9	212

### 3.2 Friction and wear tests

**Fig. 3:**
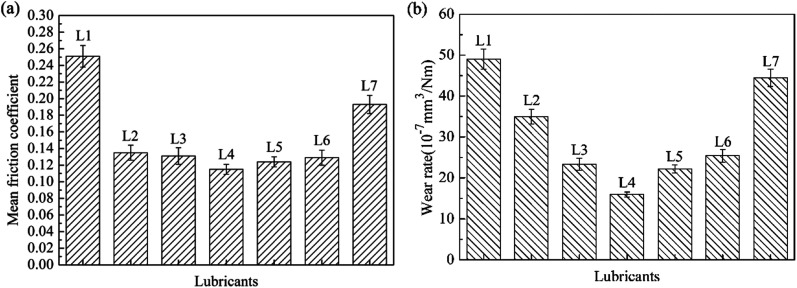
(a) Mean friction coefficient curves and (b) wear rate of 150SN and prepared lubricants.

Fig. 3 shows the mean friction coefficient and wear rate under the lubrications of lubricants. For L1, the mean friction coefficient was about 0.25 and the wear rate was 48.82 × 10^−7^ mm^3^ Nm^−1^. For L2, the mean friction coefficient and wear volume were both remarkably reduced to 0.13 and 35.06 × 10^−7^ mm^3^ Nm^−1^ respectively. Compared with L2, the mean friction coefficient and wear rate for oil with all attapulgite/La_2_O_3_ nanocomposites were all further reduced. The mean friction coefficient and wear rate were the lowest when the concentration of La_2_O_3_ is 0.4 wt%. In the effect of attapulgite/La_2_O_3_ nanocomposite containing 0.4 wt% La_2_O_3_, the friction coefficient and wear loss for 150SN were reduced by 55.20% and 64.38%. Thereby, it is concluded that the as-prepared attapulgite/La_2_O_3_ nanocomposite have better friction-reducing and anti-wear properties than the single attapulgite and La_2_O_3_ lubricant additives. During the friction procedure, attapulgite/La_2_O_3_ nanocomposites were delivered into the worn area along with the flow of lubricating oil. Attapulgite nanofibers and La_2_O_3_ nanoparticles were absorbed on the rubbing surface at the same time. Hence there is a competition between the two kinds of nanoparticles. When the content of La_2_O_3_ is lower than 0.4 wt%, the function of La_2_O_3_ cannot be fully exploited. While the content of La_2_O_3_ is too high, the repairing effect of attapulgite is inhibited. So the optimum content of La_2_O_3_ is 0.4 wt%.

**Fig. 4:**
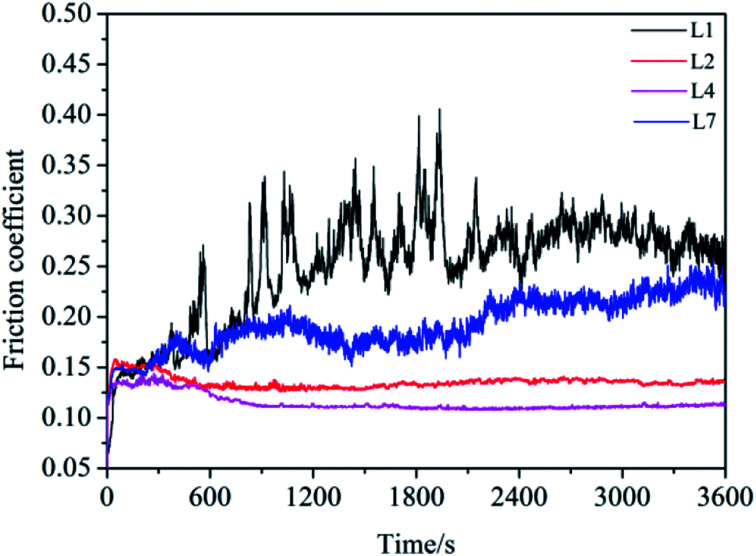
The friction coefficient curve of L1, L2, L4 and L7 with sliding time.

The curves of friction coefficient with sliding time were shown in Fig. 4. For L1 and L7, the friction coefficient displayed high value coupled with much fluctuation. This demonstrated that the lubricating film of 150SN cannot afford the high pressure between the friction pairs. And the strengthening effect of the single La_2_O_3_ powder is not obvious. For L2 and L4, after a short time of running-in process, the curves kept steady to the end of tests.

### 3.3 SEM and EDS analysis of the rubbing surfaces

**Fig. 5:**
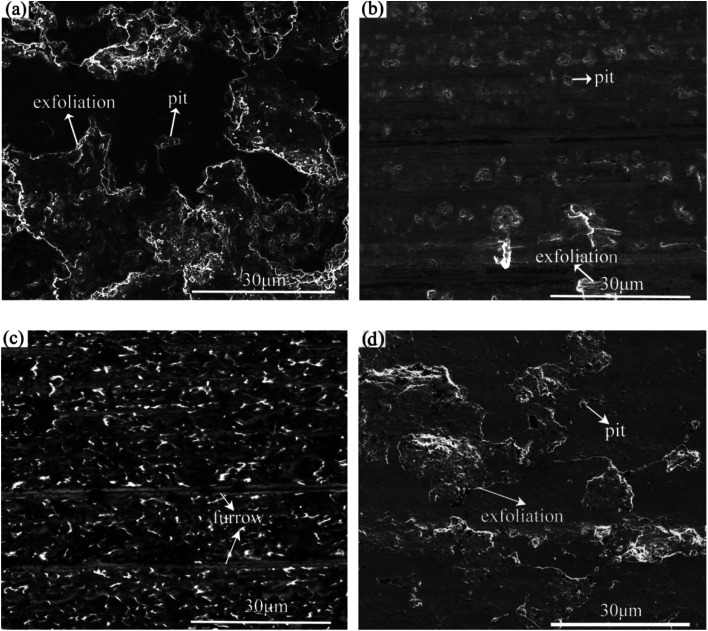
SEM images of the rubbing surfaces lubricated with (a) L1; (b) L2; (c) L4; (d) L7.

Fig. 5 shows SEM images of the rubbing surfaces lubricated with L1, L2, L4 and L7. For L1 and L7, some deep pits and large area material exfoliations were observed on the rubbing surface, indicating serious fatigue-exfoliative wear. For L2, only a little pits and area exfoliations existed on the rubbing surface, indicating that the wear was slight. The rubbing surface for L4 did not show any obvious signs of wear, a smooth and flat tribofilm was formed on the rubbing surface. Moreover, some shallow furrows were observed on the rubbing surface, may be attributed to the rolling effect of La_2_O_3_ nanoparticles.

**Fig. 6:**
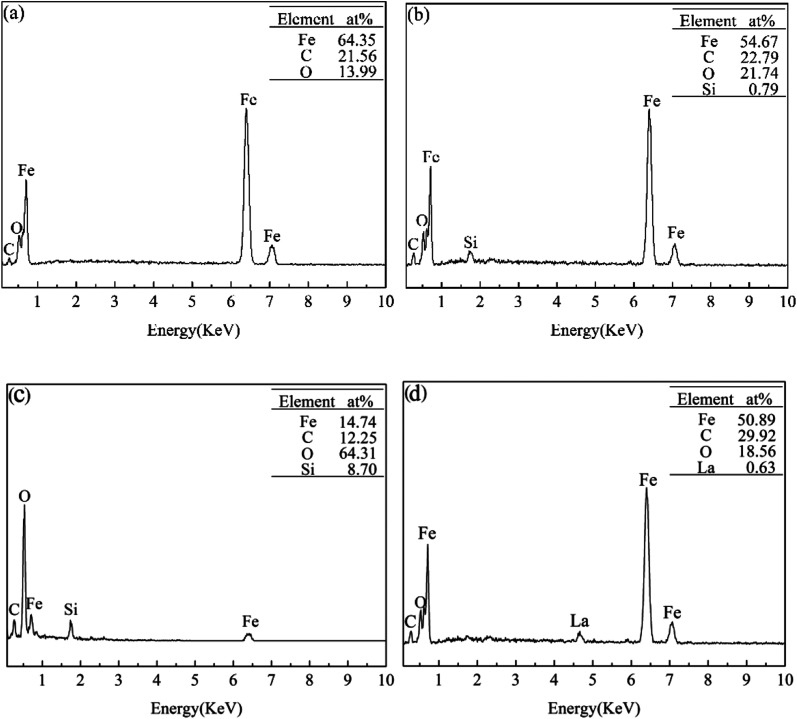
EDS patterns of the rubbing surfaces lubricated with (a) L1; (b) L2; (c) L4; (d) L7.

Fig. 6 shows the EDS analysis results of the rubbing surfaces shown in Fig. 5. For L1, Fe, C and O existed on the rubbing surface. The O element comes from the air. For L2, Fe, C, O and a small amount of Si could be detected on the rubbing surface. Moreover, compared with L1, there is an increased content of O and a decreased content of Fe. For L7, Fe, C, O and a small amount of La existed on the rubbing surface. For L4, Fe, C, O and Si existed on the rubbing surface. No La was detected. This suggested that the adsorbability of La_2_O_3_ nanoparticles on the tribofilm surface is poor. Compared with L2, the content of Fe decreased from 54.67% to 14.74%, the content of O increased from 21.74% to 64.31%, the content of Si increased from 0.79% to 8.70%. Under the lubrication of the base oil, asperity sheared together when the friction pairs moved. Plastic deformation occurred on the metal surface, accelerating the diffusion of oxygen into the metal. Thus the oxide layer was formed. In the effect of attapulgite, tribochemical reactions occurred on the rubbing surface, a tribofilm enriched in Fe, C, O, Si was formed. Under the lubrication of oil with attapulgite/La_2_O_3_ nanocomposite, the tribochemical reaction between attapulgite and friction pairs were promoted by La_2_O_3_, a tribofilm with higher content of O and Si was formed.

### 3.4 Three-dimensional morphologies of the rubbing surfaces

**Fig. 7:**
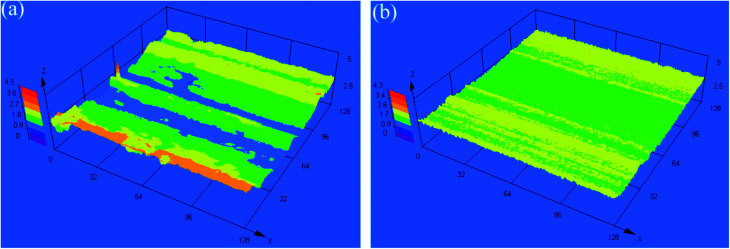
Three-dimensional morphologies of the rubbing surfaces for (a) L1 and (b) L4.

Fig. 7 shows the three-dimensional morphologies of the rubbing surfaces for L1 and L4. For L1, the rubbing surface was very course, with some high peaks and deep troughs. As for L4, the rubbing surface was smooth and flat, no obvious peaks and troughs appeared. This result agreed with the tribological test results well.

### 3.5 XPS analysis of the rubbing surfaces

**Fig. 8:**
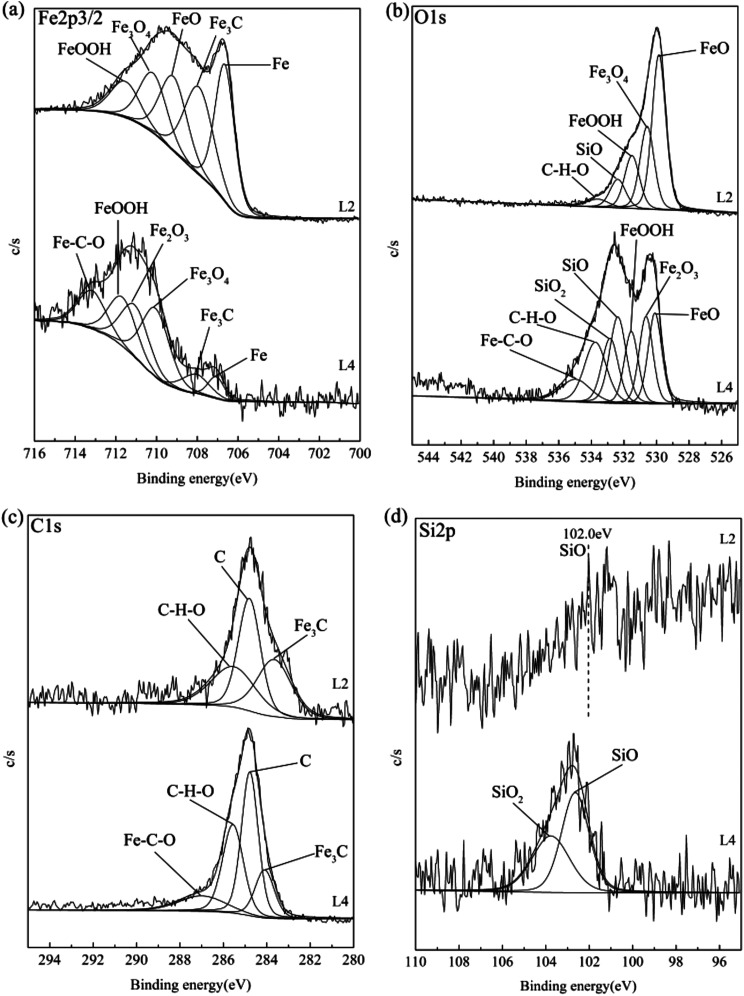
XPS analysis results of the rubbing surfaces for 150SN with attapulgite powder (L2) and attapulgite/La_2_O_3_ nanocomposite (L4): (a) Fe2p3/2; (b) O1s; (c) C1s; (d) Si2p.

Fig. 8 shows the XPS analysis results of the rubbing surfaces for 150SN with attapulgite powder (L2) and attapulgite/La_2_O_3_ nanocomposite (L4). Fe2p3/2 spectra for L2 was fitted into five sub-peaks at binding energy at 706.7 eV (Fe), 708.1 eV (Fe_3_C), 709.1 eV (FeO), 710.1 eV (Fe_3_O_4_) and 711.5 eV (FeOOH). While for L4, Fe2p3/2 spectra could be fitted into 707.0 eV (Fe), 708.1 eV (Fe_3_C), 710.1 eV (Fe_3_O_4_), 711.0 eV (Fe_2_O_3_), 711.5 eV (FeOOH) and 713.1 eV (Fe–C–O).^10,16,17^ In addition, compared with L2, the peak area of Fe and Fe_3_C both obviously decreased, and the peak areas of iron oxides remarkably increased. This result consistent with the EDS results completely. O1s spectra for L2 could be fitted into five peaks with binding energy at 529.8 eV, 530.3 eV, 531.7 eV, 532.5 eV and 533.8 eV, which correspond to FeO, Fe_3_O_4_, FeOOH, SiO and organic compound (C–H–O). As for L4, O1s spectra was fitted into FeO (530.1 eV), Fe_2_O_3_ (530.5 eV), FeOOH (531.7 eV), SiO (532.5 eV), SiO_2_ (533.0 eV) and organic compound (533.8 and 534.9 eV).^10,16–18^ For L2 and L4, the C1s spectra were both fitted into three peaks corresponding to Fe_3_C, C and organic compound.^10,16^ For L2, the Si2p peak at 102.0 eV was correspond to SiO. The Si2p spectra for L4 could be divided into two sub-peaks at the binding energy of 102.0 eV (SiO) and 103.8 eV (SiO_2_).^10,18^

From XPS analysis results, it was concluded that a tribofilm mainly composed of Fe, Fe_3_C, FeO, Fe_3_O_4_, FeOOH, SiO and organic compound was formed on the rubbing surface of 150SN with attapulgite powder. Fe, Fe_3_C are the main components of the matrix, indicating some abrasive particles and wear debris of metal were melted into the tribofilm. For 150SN with attapulgite/La_2_O_3_ nanocomposite, there is also a formation of tribofilm, which was mainly consisted with Fe, Fe_3_C, Fe_3_O_4_, Fe_2_O_3_, FeOOH, SiO, SiO_2_ and organic compound. In addition, in the effect of La_2_O_3_, the contents of iron oxides and silicon oxides were obviously increased.

### 3.6 Nano-indentation test for the rubbing surfaces

**Fig. 9:**
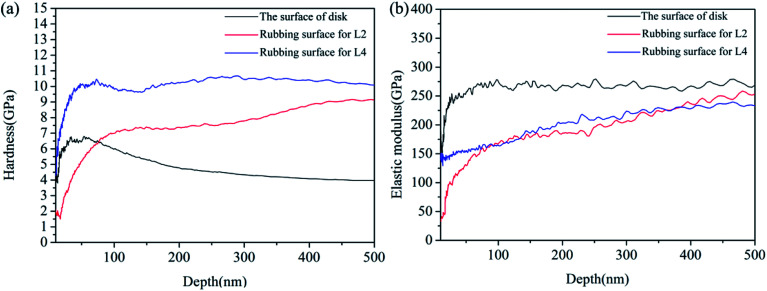
(a) Hardness–displacement curves and (b) elastic modulus–displacement curves of the disk and rubbing surfaces.

Nano-indentation test results for the steel disk and rubbing surfaces of L2 and L4 are shown in Fig. 9 and Table 3. The hardness–displacement curves were shown in Fig. 9(a). For the steel disk, the hardness decreased gradually from 6 GPa to 4 GPa with the increase of the pressed depth. For the tribofilm formed on the rubbing surface of L2, the hardness increased gradually from 7 GPa to 8 GPa. While for the tribofilm formed on the rubbing surface of L4, the hardness kept stable about 10 GPa. The elastic modulus–displacement curves were shown in Fig. 9(b). For the steel disk, the elastic modulus kept stable about 250 GPa. For the tribofilm formed with L2 and L4, the elastic modulus both increased gradually from 150 GPa to 230 GPa with the increase of the pressed depth. During the friction procedure, the deposited lubricating additives took tribochemical reactions with the rubbing surfaces, leading to a formation of tribofilms. The thickness and compactness of the tribofilm increased constantly. Thus the mechanical properties of the tribofilm were improved with the increase of the pressed depth.

Table 4 shows the average hardness (*H*), average elastic modulus (*E*) and *H*/*E* ratio of the steel disk and tribofilms. For the steel disk, the hardness and elastic modulus was 4.30 GPa and 268.01 GPa. For the tribofilm formed by L2, the hardness and the elastic modulus was 8.15 GPa and 216.21 GPa. AS for the tribofilm formed by L4, the hardness increased to 10.41 GPa and the elastic modulus was 221.49 GPa. It is concluded that the tribofilms possess higher hardness and a little lower elastic modulus than the AISI 1045 steel. So the tribofilms possess some excellent properties of metal and ceramics meanwhile, such as high hardness, high wear resistance, good ductility and good plasticity. It is reported that the *H*/*E* ratio can directly reflect the wear resistance of the materials.^19^ A material with a higher *H*/*E* ratio is easier to recover from elastic deformation induced by external stress, thus fewer asperities would formed, resulting in a lower friction and wear. It can be seen from Table 4 that the *H*/*E* value of the metal disk was the lowest and that for L4 was the highest. This result further demonstrated that the tribofilms generated on the rubbing surfaces by L2 and L4 possess excellent mechanical properties, especially L4.

**Table 4:** Average hardness and modulus of elasticity of the disk and rubbing surfaces

	Nano-mechanical properties		
	*H* (GPa)	*E* (GPa)	*H*/*E*
Steel disk	4.30	268.01	0.016
Rubbing surface for L2	8.15	216.21	0.038
Rubbing surface for L4	10.41	221.49	0.047

### 3.7 Discussion

**Fig. 10:**
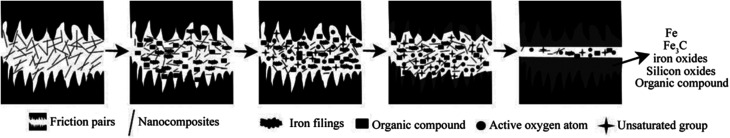
Formation mechanism of the tribofilm generated by attapulgite/La_2_O_3_ nanocomposite.

From the experimental results, we can see that the as-prepared attapulgite/La_2_O_3_ nanocomposite displayed good friction-reducing and antiwear properties. In the effect of nanocomposite, a tribofilm composed of Fe, Fe_3_C, iron oxides, silicon oxides and organic compound with high hardness was formed on the rubbing surface. The formation mechanism of the protective film is mainly associated with the friction thermodynamics effect and unique crystal structure of attapulgite. The formation mechanism of the tribo-chemical film was schematically depicted in Fig. 10. In the rubbing process, attapulgite/La_2_O_3_ nanoparticles were adhered onto the rubbing surface. Under the action of extrusion pressure, shearing force and friction, interlayer cleavage, structural water removal and lattice distortion occurred, leading to structural instability of attapulgite, the crystallinity of attapulgite decreases and tend to disordering. In the meantime, a plenty of active oxygen atoms, free bonds (Si–O–Si, O–Si–O) and hydrogen bond released.^9,10^ In the effect of high flash temperature and high shear stress between the friction pairs, such released substances took tribo-chemical reactions with the metallic matrix, and La_2_O_3_ acted as a catalyst to accelerate the tribo-chemical reactions.^20^ The main tribo-chemical reactions were as follows: (1) the active iron atoms on the rubbing surface and iron filings took reaction with active oxygen atoms and hydroxyl groups released by attapulgite, forming multiphase iron oxides and FeOOH. (2) The Si–O–Si and O–Si–O active groups recombine to form silicon oxides. (3) Part of the 150SN cracked and reacted with matrix to form organic compounds. The tribo-chemical reaction products and melted iron chips were squeezed together by contact pressure and friction force, then amorphous tribofilm was formed. The tribofilm and matrix combined together by metallurgical bonding. With the prolongation of sliding time, the tribofilm became thicker and denser. Meanwhile, part tribofilm were worn out. At last, dynamic equilibrium between the forming and worn of tribofilm, consequently stable friction coefficient presented. The tribofilm could prevent the metallic friction pairs from direct contacting, greatly relieved the adhesion effect between the friction pairs. The tribofilm possess high hardness, which could bring milder distortion and better wear resistance. In addition, the tribofilm possess good plasticity and ductility like metal. The inclination of forming microcracks and peeling during the friction procedure is greatly reduced. Hence, the attapulgite/La_2_O_3_ nanocomposite could remarkably improve the friction-reducing and antiwear properties of 150SN base oil.

**Fig. 11:**
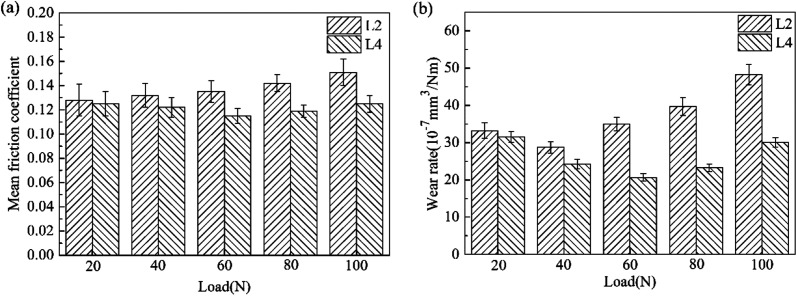
The effect of load on the tribological properties of lubricants.

In order to investigate the tribological behavior of attapulgite/La_2_O_3_ nanocomposite more systematically, the effect of load and frequency on the friction coefficient and wear rate was investigated. Fig. 11 shows the effect of load on the mean friction coefficient and wear rate respectively for L2 and L4. Compared with L2, the mean friction coefficient and wear rate for L4 were lower at all test loads, especially at 60 N, 80 N and 100 N. This result may be due to following reason: the higher the load is, the higher the generated heat will be. Thus the catalysis of La_2_O_3_ is more significant. In addition, for L4, the mean friction coefficient and wear rate were both the least at 60 N. This result may be attributed to the competition between the formation and abrasion of the tribofilm. With the increase of the load, more energy is supplied to impel the reaction between the nanoparticles and metallic matrix. But when the load is too high, the abrasion of the tribofilm may be dominated.

**Fig. 12:**
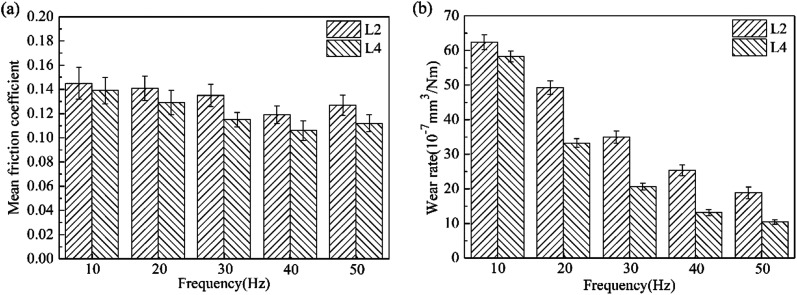
The effect of frequency on the tribological properties of lubricants.

Fig. 12 shows the effect of frequency on the mean friction coefficient and wear rate for L2 and L4. In the presence of La_2_O_3_, the mean friction coefficient and wear rate for L2 both decreased at all test frequencies. Moreover, with the increase of the frequency, the mean friction coefficient and wear rate for L2 and L4 both decreased continuously. Under the lubrication of the lubricating oil, the lubrication regime is mixed lubrication. From the Stribeck curves,^21^ it can be seen that the friction coefficient under mixed lubrication is proportional to speed and inversely proportional to load. Under the same load, the friction and wear both decreased along with the increase of frequency.

From the tribological tests, it can be concluded that La_2_O_3_ nanoparticles can improve friction-reducing and antiwear properties of the oil containing attapulgite. However, the improvement effect on the friction-reducing property is not significant. This phenomenon can be explained as follows: under the lubrication of oil containing attapulgite and oil containing attapulgite/La_2_O_3_ nanocomposite, the chemical composition of the tribofilms were basically the same, hence the self-lubrication properties of the formed tribofilms were not obviously different. In the other hand, in the effect of La_2_O_3_ nanoparticles, the thickness, uniformity and compactness of the tribofilm were obviously improved, consequently the antiwear property of oil containing attapulgite was remarkably improved.

## 4. Conclusions

The tribological properties of attapulgite/La_2_O_3_ nanocomposite as lubricant additive were investigated. In the effect of attapulgite/La_2_O_3_ nanocomposite, the friction-reduction and anti-wear properties of the oil were obviously improved. During the friction procedure, tribochemical reactions between attapulgite powders and metal matrix occurred, and La_2_O_3_ can be served as catalysts to accelerate the tribochemical reactions. At last, a tribofilm mainly composed of Fe, Fe_3_C, FeO, Fe_2_O_3_, FeOOH, SiO, SiO_2_ and organic compound was formed on the rubbing surface. The multiphase tribofilm possess excellent self-lubricating ability, antiwear property and mechanical properties, which is responsible for the reduction of friction and wear. We expect our work can offer new routes to develop self-repairing lubricant additives with high friction-reduction and antiwear properties.

## Conflicts of interest

There are no conflicts to declare.

## Supplementary Material
